# Long-term culture of patient-derived pheochromocytoma organoids

**DOI:** 10.3389/fendo.2026.1781556

**Published:** 2026-03-18

**Authors:** Marit F. van den Berg, Elpetra P. M. Timmermans-Sprang, Jan Zethof, Benno Kusters, Andre Olthaar, Antonius E. van Herwaarden, Monique E. van Wolferen, Henri J. L. M. Timmers, Hans S. Kooistra, Margo Dona, Sara Galac

**Affiliations:** 1 Department Clinical Sciences, Faculty of Veterinary Medicine, Utrecht University, Utrecht, Netherlands; 2 Department of Plant and Animal Biology, Radboud Institute for Biological and Environmental Sciences (RIBES), Radboud University, Nijmegen, Netherlands; 3 Department of Pathology, Radboud University Medical Center, Nijmegen, Netherlands; 4 Department of Laboratory Medicine, Radboud University Medical Center, Nijmegen, Netherlands; 5 Department of Internal Medicine, Radboud University Medical Center, Nijmegen, Netherlands; 6 Institute of Biology Leiden, Leiden University, Leiden, Netherlands

**Keywords:** adrenal tumor, culture, organoid, personalized therapy, pheochromocytoma, preclinical model

## Abstract

**Introduction:**

Pheochromocytomas (PCCs) are rare neuroendocrine tumors with limited treatment options once metastasized. Progress toward effective therapies has been hindered by their rarity, disease heterogeneity, and lack of representative preclinical models. Organoids are three-dimensional, self-renewing structures that recapitulate key features of their tissue of origin, providing valuable platforms for disease modeling, drug screening, and personalized medicine. This study aimed to establish and characterize patient-derived organoid cultures of PCCs.

**Methods:**

Tumor tissue from two patients undergoing surgery for PCC was collected to generate organoid cultures. Organoids were expanded under growth factor-enriched conditions and characterized by histology, immunohistochemistry, immunofluorescence, gene expression via qPCR, and metanephrine quantification via LC-MS/MS.

**Results:**

Both cultures could be maintained for multiple weeks and serially passaged, with PCC1 maintained in culture for 129 days and expanded up to passage 3 (passaged every 34–53 days), and PCC2 cultured for 139 days, reaching passage 2 (passaged every 63 days). At the protein level, organoids expressed both stem/progenitor markers (nestin, vimentin, SOX10) and chromaffin differentiation markers (synaptophysin, CD56), although expression of the latter was markedly reduced compared to primary tumor tissue. Metanephrine production confirmed initial secretory activity, but hormone production declined over time.

**Conclusions:**

This pilot study demonstrates that patient-derived organoid cultures can be established from human PCCs and maintained with serial passaging. In early stages, these organoids maintain key phenotypic and functional traits of their original tumors; however, these features diminish over time, consistent with a progressive loss of chromaffin differentiation and/or expansion of less differentiated cell populations. Further optimization is required to improve their long-term proliferation and differentiation potential. Together, these findings provide a proof-of-concept for the development of patient-derived PCC organoid models, which may ultimately serve as a platform for studying PCC biology and for future exploration of personalized therapeutic approaches.

## 1 Introduction

Pheochromocytomas (PCCs) are rare neuroendocrine tumors originating from chromaffin cells of the adrenal medulla, typically associated with excess catecholamine production (1). Together with extra-adrenal paragangliomas (PGLs), they are classified as pheochromocytoma and paraganglioma (PPGL), a group of tumors with the highest rate of heritability among all tumor types (2, 3). PPGLs are highly heterogeneous, with over 20 identified susceptibility genes (2). Based on underlying genetic alterations and disrupted signaling pathways, PPGLs are categorized into three main molecular clusters: pseudohypoxia-related, kinase-signaling related, and Wnt-signaling related (2). While surgical resection remains the treatment of choice for localized, non-metastatic PCCs, therapeutic options for patients with metastatic disease are limited and prognosis is poor, with an overall mortality rate of 53% (4, 5).

Progress in therapy development is hindered by the lack of representative and scalable preclinical models (6–8). Attempts to establish human PCC cell lines have not been successful to date, with all reported cultures ultimately being lost over time, presumably due to limited proliferative capacity (6, 8–10). Patient-derived xenograft models can preserve key genetic features of the original tumor; however, successful engraftment is variable and tumor formation typically requires several months, raising concerns regarding scalability and ethical considerations related to animal use (11, 12). While genetically engineered zebrafish, mouse, and rat models have been developed to mimic specific genetic alterations observed in human PCCs (13–15), they exhibit substantial interspecies differences and typically represent a single genetic background at a time, making it challenging to capture the broad genetic and phenotypic heterogeneity of PCCs (6, 7). In addition, several three-dimensional adrenal culture systems have been developed to model adrenal cortex–medulla interactions or adrenal progenitor cell biology, including spheroid and “adrenoid” models. However, these approaches are based on murine PCC cell lines (16) or normal adrenal tissue [including both non-human and human sources (17–20)], and therefore do not recapitulate key aspects of human PCC biology. More recently, patient-derived primary cultures of human PCCs have enabled personalized drug screening (21–23); however, they have significant limitations: they reflect only short-term responses, with culture periods of approximately 72 hours, followed by drug incubation for an additional 72 hours. Moreover, they lack the complexity of the tumor microenvironment, and require regeneration for each patient, limiting their scalability and translational value (23, 24).

Patient-derived organoids have emerged as a promising model in other tumor types. These three-dimensional, self-renewing cultures derived from patient tumor cells often comprise stem/progenitor and differentiated cell populations and can closely recapitulate the architecture, cellular heterogeneity, and functionality of their tumor of origin. They allow long-term expansion and provide a powerful translational platform for disease modeling, drug screening, and personalized approaches (25, 26). In addition, organoid biobanks capturing genetic diversity can link tumor mutations to treatment response (26). Against this background, patient-derived three-dimensional tumor culture systems, referred to as tumor organoids, have recently been applied to human PPGLs to enable rapid, short-term ex vivo assessment of therapeutic responses (27). However, it remains unclear to what extent such cultures can be maintained over longer periods with serial passaging and how cellular identity and functional characteristics evolve during prolonged *in vitro* culture. In parallel, we have established long-term organoid cultures from both normal canine adrenal medulla and canine PCCs, demonstrating the feasibility of sustained organoid culture in another species with naturally occurring PCC (28). Whether a comparable long-term organoid-based approach can be achieved using human PCC tissue has not yet been investigated.

In this pilot study, we aimed to generate and characterize organoid cultures derived from human PCCs as a proof-of-concept toward the development of a representative *in vitro* model to study PCC biology and support therapeutic development.

## 2 Materials and methods

### 2.1 Tumor tissue collection and processing

Tumor tissue was collected from patients with PCC undergoing adrenalectomy at Radboud University Medical Center. Written informed consent was obtained from both patients for the use of their tumor tissue and associated clinical data for research and publication purposes, after ethical approval from the institutional research board (METC Oost-Nederland: 2023-16451). A representative portion of the tumor was transported on ice in Dulbecco’s Modified Eagle Medium (DMEM)/F-12 (Gibco, New York, USA) with 10% (v/v) fetal calf serum (FCS; Gibco) and 1% (v/v) penicillin/streptomycin (P/S; 10,000 U/mL; Gibco) to the Faculty of Veterinary Medicine, Utrecht University. Upon arrival, within a few hours after surgical removal, tumor fragments were mechanically and enzymatically dissociated to single cells, followed by red blood cell lysis if needed, and single cells were subsequently counted. A detailed description of the tissue processing and digestion protocol is available in **Supplementary File 1**.

### 2.2 Organoid culture

Single cells were resuspended in Cultrex^®^ basement membrane extract (BME; R&D Systems, Minneapolis, MN, USA) and seeded as 15 μL droplets onto pre-warmed 48-well plates. After gelation, droplets were overlaid with pre-warmed expansion medium (EM). EM optimized for canine adrenal tissue was used and consisted of Advanced DMEM/F-12 (Gibco), supplements (GlutaMAX, HEPES, P/S, B27, N2, N-acetylcysteine, calcium gluconate), and a growth factor combination (WREFLD: Wnt, R-spondin-3, EGF, FGF2, LIF, DHEAS) (28). As PCCs can express high levels of IGF2 and IGF signaling has been implicated in chromaffin cell proliferation and survival (29, 30), both tumors were cultured in parallel in WREFLD with or without IGF2. Cultures were maintained at 37 °C in a humidified incubator with 5% CO_2_, and the medium was refreshed 2–3 times per week. Full details on the expansion medium composition, growth factor concentrations, and culture conditions are provided in **Supplementary File 1**.

### 2.3 Organoid passaging

To assess the ability to maintain and passage cultures over time, organoids and dense cellular clusters were dissociated using accutase (Gibco), with additional collagenase (Sigma-Aldrich, Merck KGaA, Darmstadt, Germany) treatment when required. Dissociated cells were combined, resuspended in medium supplemented with Y-27632 (AbMole BioScience, Houston, USA), and replated in BME droplets. Pre-warmed medium containing 40% (v/v) conditioned medium from the previous passage and 60% (v/v) fresh EM with WREFLD, with or without IGF2, was added. Full details of the passaging protocol are provided in **Supplementary File 1**.

### 2.4 Organoid differentiation

To induce differentiation, PCC organoid cultures were exposed to 10 µM dexamethasone (Dex; Sigma-Aldrich) and/or 100 nM phorbol 12-myristate 13-acetate (PMA; Millipore, Billerica, MA, USA), either in the presence or absence of the growth factor cocktail WREFLD.

### 2.5 Metanephrine measurements

We quantified metanephrine, normetanephrine, and 3-methoxytyramine concentrations in conditioned medium at different time points from both PCC organoid cultures by liquid chromatography-tandem mass spectrometry (LC-MS/MS) after derivatization with propionic anhydride [adapted from Faassen et al., 2020 (31)] and solid-phase extraction (Oasis HLB, Waters Corporation, Milford, MA, USA). Each measurement was performed in duplicate, with each duplicate comprising a pooled sample from six wells. Because continuous culture precluded cell lysis for protein measurement, we standardized sample input by using the same number of wells rather than normalizing to protein content. Expansion medium supplemented with growth factors served as a negative control. Samples were stored at –70 °C (up to 14 months), shipped on dry ice to the Department of Laboratory Medicine, Radboud University Medical Center, and held at –80 °C until analysis within two months of receipt.

### 2.6 Marker selection for organoid characterization

To characterize the cellular composition of the organoids, a panel of markers was selected based on prior studies. Markers commonly used to identify stem/progenitor cells in the adrenal medulla included SRY-box transcription factor 10 (SOX10), vimentin (VIM), and nestin (NES) (19, 20, 32–34). Although glial fibrillary acidic protein (GFAP) is primarily used to label sustentacular cells, we also used it to examine the potential progenitor role of these cells (32, 34). Additionally, we focused on chromogranin A (CHGA), synaptophysin (SYP), CD56, and phenylethanolamine N-methyltransferase (PNMT) as chromaffin cell markers, and tubulin beta 3 class III (TUBB3) as a neural marker (35, 36). Tyrosine hydroxylase (TH) served as a combined marker for both chromaffin cells and catecholaminergic neurons. Proliferative activity was evaluated using Ki67.

### 2.7 Histology and immunohistochemistry

Organoids, dense cellular clusters, and primary tumors were fixed in 4% (v/v) buffered paraformaldehyde (PFA), paraffin-embedded, and cut into 4 μm sections. Sections were deparaffinized, rehydrated, and processed for immunohistochemistry (IHC) using standard protocols. Hematoxylin and eosin staining, CHGA, and Ki67 IHC of the primary tumor tissue were performed as part of routine diagnostic pathology following surgery. Additional immunohistochemical stainings on primary tumor tissue (SYP, CD56, SOX10, NES, and VIM) were performed as part of the present study. Sections underwent antigen retrieval, blocking, and incubation with primary and secondary antibodies. Positive and negative controls were included. Full staining protocols, including antigen retrieval conditions and antibody dilutions, are detailed in **Supplementary File 1** and **Supplementary Table 1**.

### 2.8 Immunofluorescence staining and imaging

Cell suspensions mixed with BME were plated as 30 μL droplets onto pre-warmed 4-compartment CellView™ cell culture dishes (Greiner Bio One, Frickenhausen, Germany) and cultured until organoid formation. Organoids were fixed in 3% (v/v) PFA supplemented with 0.1% (v/v) glutaraldehyde and stored in 75% (v/v) ethanol at 4 °C prior to staining. Organoids were washed, quenched, permeabilized, and blocked before staining with primary antibodies, followed by fluorescently labeled secondary antibodies and DAPI nuclear counterstaining. Images were acquired using an Olympus SpinSR10 spinning disk confocal microscope equipped with a 30x silicone oil immersion objective. Organoids were imaged at the mid-plane of their height. For detailed staining protocols, full acquisition parameters, and antibody information and incubation conditions, see **Supplementary File 1** and **Supplementary Table 2**. Negative control organoids were stained with secondary antibodies only (Alexa Fluor™ 488 anti-rabbit and Alexa Fluor™ 568 anti-mouse), which were used consistently across all experiments. Fluorescence intensities were normalized to the mean background signal of these negative controls to calculate fold-over-background values.

### 2.9 RNA isolation and quantitative real-time RT-PCR

RNA was isolated from PCC tumor tissue, cell suspensions, organoids, and dense cellular clusters at different passages using the RNeasy Mini Kit (Qiagen, Hilden, Germany). To ensure sufficient RNA yield, samples cultured under WREFLD and WREFLD + IGF2 conditions were combined. Additional RNA was collected from PCC1 after the differentiation experiment at P3. RNA concentration and purity were assessed, and cDNA was synthesized. Quantitative real-time RT-PCR (qPCR) was performed to assess the expression of adrenomedullary markers (*CHGA*, *SYP*, *TH*, *PNMT*), stem/progenitor markers (*NES*, *VIM*, *SOX10*, *GFAP*), and neural markers (*TUBB3*). Candidate reference genes were evaluated, and tyrosine 3-monooxygenase/tryptophan 5-monooxygenase activation protein zeta (*YWHAZ*), glucuronidase beta (*GUSB*), and hypoxanthine phosphoribosyltransferase (*HPRT1*) were selected based on expression stability across samples using the geNorm method (37). Relative gene expression was calculated using the 2^–ΔΔCt method (38). For details on primer design and validation, and reference gene selection, see **Supplementary File 1** and **Supplementary Table 3**.

### 2.10 Statistical analysis

Statistical analyses were performed using GraphPad Prism software (version 10.1.1; GraphPad Software Inc. Dotmatics, Boston, MA, USA). Data were first tested for normality using the Shapiro‐Wilk test. Gene expression levels between groups (PCC tissue, cell suspensions, organoids at passages P0, P1, and P2, and dense cellular clusters at passages P0, P1, and P2) were compared using the Kruskal-Wallis test. The primary tumor tissue served as the reference group for all comparisons. The Friedman test, which accounts for matched data, could not be applied due to missing values in some conditions (e.g., absence of organoids and dense cellular clusters from PCC2 at P2). In the differentiation experiment, gene expression levels were compared between culture conditions, using WREFLD as the reference group. These comparisons were also performed using the Kruskal–Wallis test. For all analyses, when significant differences were detected, *post hoc* analyses were performed using Dunn’s correction for multiple comparisons. A *P* value of <0.05 was considered statistically significant.

## 3 Results

### 3.1 Establishment of patient-derived pheochromocytoma organoids

**Figure 1:**
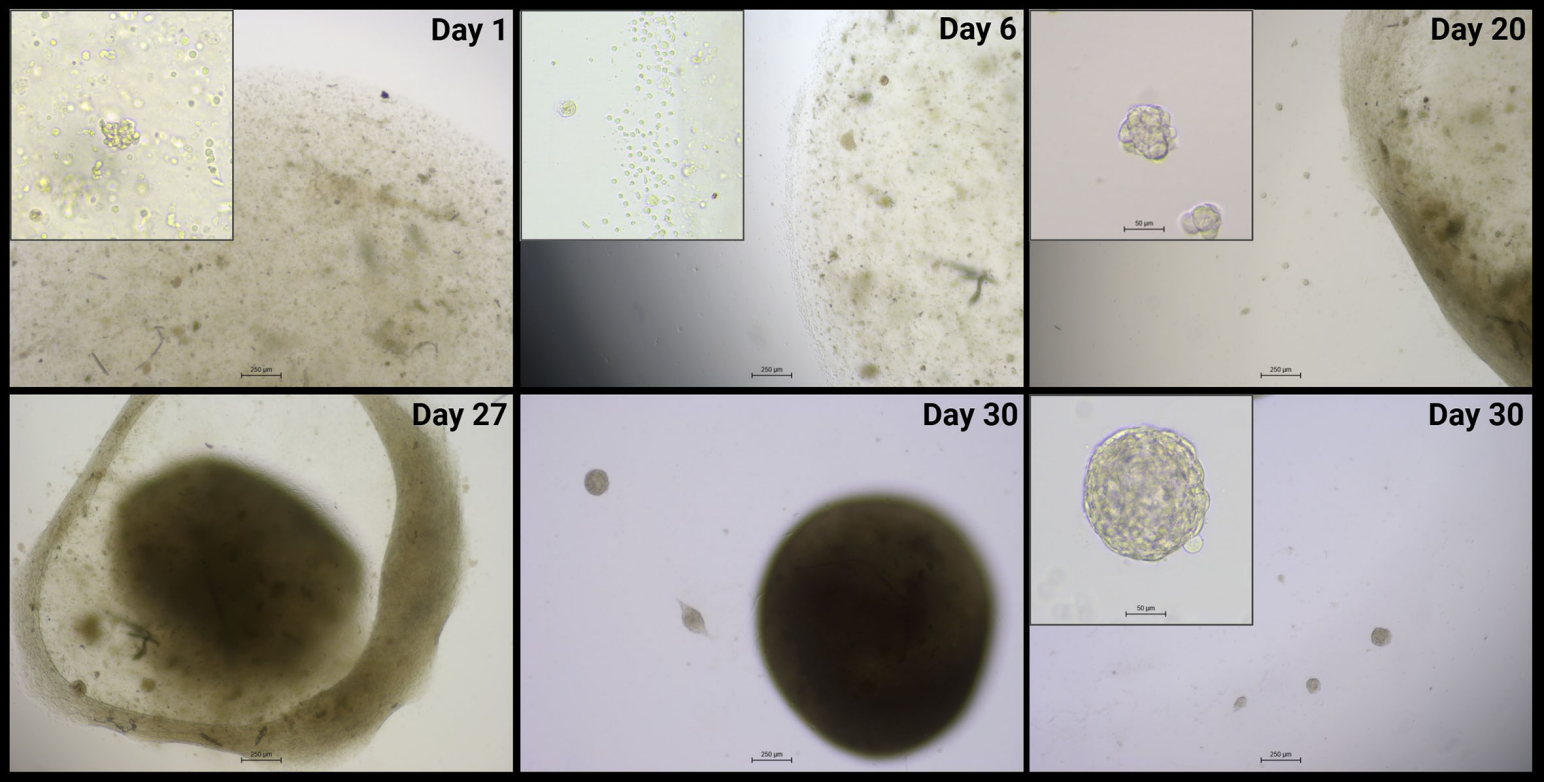
Representative bright-field images (4× magnification; 20× insets) of PCC1 depict organoid formation from initial plating of a cell suspension in a BME droplet through to passaging. On day 1, individual chromaffin cells and small aggregates are visible within the BME droplet. By day 6, these clusters have enlarged and some cells exhibit elongated, varicose extensions, while a few rounded cells begin to migrate beyond the droplet boundary. As culture progresses, clusters within the BME droplet expand further and interconnect into a web-like network by day 20, with cells in this network displaying a mesenchymal-like morphology. This network then contracts into a dense, compact cellular cluster by days 27–30. Small organoids emerge outside the BME droplet around day 14 and increase in number and size over time, as illustrated by the day 20 and day 30 images. By day 30, both organoids and dense cellular clusters are prominent. Images shown are from PCC1; PCC2 followed a comparable developmental trajectory, albeit at a slower pace, and is therefore not shown separately. Scale bars: main panels 250 µm; insets (where shown) 50 µm. PCC, pheochromocytoma; BME, basement membrane extract.

To generate PCC organoid cultures, excess PCC tissue not required for clinical diagnostics was collected from two patients undergoing adrenalectomy. Clinical and pathological details from both patients are summarized in **Table 1**. Both patients had undergone routine clinical germline genetic screening for known PCC susceptibility genes, which did not identify any pathogenic variants. **Figure 1** shows the progression through different stages of PCC organoid development. Both PCC1 and PCC2 could be maintained in culture over multiple months and could be serially passaged. PCC1 exhibited more robust growth, with successful expansion to passage 3 over a total of 129 days in culture. Passage intervals for PCC1 ranged from 34 to 53 days (P0–P1: 34 days; P1–P2: 53 days; P2–P3: 41 days), after which the culture was terminated for downstream qPCR analysis. In contrast, PCC2 progressed more slowly and produced fewer organoids overall; it reached passage 2 over 139 days with a consistent passage interval of 63 days (P0–P1: 63 days; P1–P2: 63 days). This culture was lost to fungal contamination shortly after passaging to P2. Parallel cultures maintained with or without IGF2 did not show consistent differences in organoid yield, size, or overall growth behavior.

**Table 1:** Clinical and pathological data from patients.

Case	Sex, age	Tumor side and size	Clinical symptoms	Pre-operative plasma metanephrines	Genetic testing	Histopathology
PCC1	Female 61 y	Left adrenal 61x60x66 mm	Paroxysmal symptoms	p3MT 183 pmol/L pMN 16,661 pmol/L pNMN 6,346 pmol/L	Negative for mutations in *SDHA, SDHB, SDHC, SDHD, SDHAF2, TMEM127, MAX, VHL, FH, MDH2, RET*	PCC with classic nested (“zellballen”) growth pattern of chromaffin cells, with occasional multinucleated tumor cells, rich vascularization, and areas of hemorrhage. The surrounding adrenal cortex showed preserved layered architecture. Clear resection margins.
PCC2	Female 40 y	Right adrenal ~12 cm	Incidental finding on kidney ultrasound	p3MT 188 pmol/L pMN 45,897 pmol/L pNMN 13,349 pmol/L	Negative for mutations in *SDHA, SDHB, SDHC, SDHD, SDHAF2, TMEM127, MAX, VHL, FH, MDH2, RET, DLST, EGLN1, EPAS1, NF1, SLC25A11, SUCLG2*	PCC with nested growth pattern of chromaffin cells, with focal capsular invasion and suspected vascular invasion.

PCC, pheochromocytoma; y, years; p3MT, plasma 3-methoxytyramine; pMN, plasma metanephrine; pNMN, plasma normetanephrine; SDHA/B/C/D, succinate dehydrogenase complex subunits A/B/C/D; SDHAF2, succinate dehydrogenase complex assembly factor 2; TMEM127, transmembrane protein 127; MAX, MYC associated factor X; VHL, von Hippel–Lindau; FH, fumarate hydratase; MDH2, malate dehydrogenase 2; RET, rearranged during transfection proto-oncogene; DLST, dihydrolipoamide S-succinyltransferase; EGLN1, Egl-9 family hypoxia inducible factor 1; EPAS1, endothelial PAS domain protein 1; NF1, neurofibromin 1; SLC25A11, solute carrier family 25 member 11; SUCLG2, succinate-CoA ligase GDP-forming beta subunit.

### 3.2 Metanephrine concentrations in PCC organoid cultures

**Figure 2:**
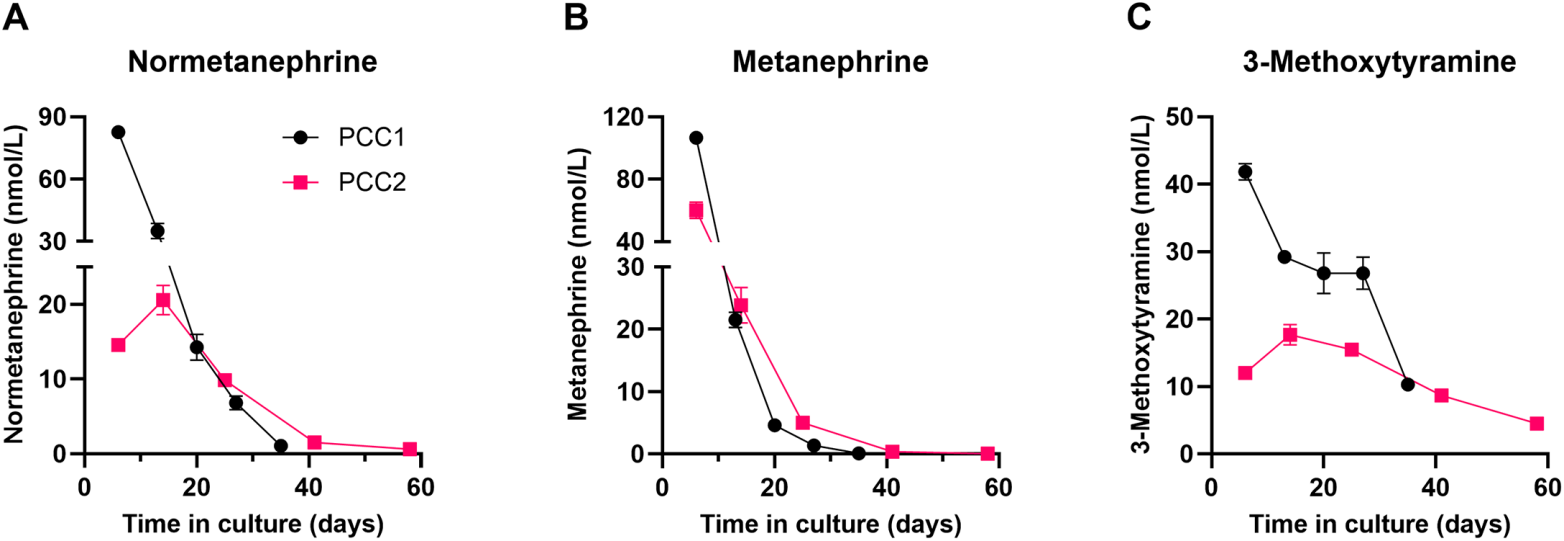
The concentrations of normetanephrine **(A)**, metanephrine **(B)**, and 3-methoxytyramine **(C)** in the cell culture supernatant were determined in duplicate by LC-MS/MS at multiple time points for both PCCs. Data are presented as mean values with error bars indicating the standard deviation. One measurement for metanephrine was below the limit of quantification (LOQ) and is displayed at the LOQ value of 0.05 nmol/L. The negative control sample showed metanephrine levels below the LOQ, while normetanephrine and 3-methoxytyramine were detectable just above the LOQ (0.02 nmol/L). However, their concentrations were markedly lower (18-fold and 144-fold lower, respectively) than those measured in the lowest culture supernatant samples.

To assess catecholamine-related secretory activity in PCC organoid cultures, metanephrine concentrations were measured in culture supernatants. For PCC1, all measurements were performed during passage 0 (P0), except for the final sample on day 35, which was taken one day after passaging to passage 1 (P1). For PCC2, all measurements were carried out during P0. Both organoid cultures showed measurable metanephrine production (**Figure 2**), with the highest concentrations observed at the early stages of culture. Metanephrine levels declined significantly over time, suggesting that while organoids initially retained key functional characteristics of their original tumors, these features were gradually lost as the cultures progressed.

### 3.3 Morphological and histological features of PCC organoids

**Figure 3:**
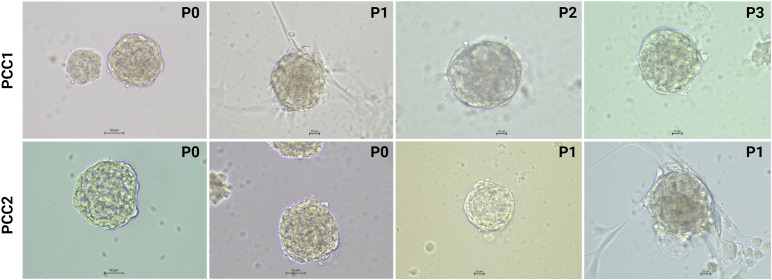
Bright-field images of human PCC organoids derived from two tumors (PCC1 and PCC2) at successive passages (PCC1: P0-P3, PCC2: P0-P1). For PCC1, images were acquired at P0 on day 33, P1 on day 50 after passaging from P0 to P1, P2 on day 38 after passaging from P1 to P2, and P3 on day 42 after passaging from P2 to P3. For PCC2, images at P0 were acquired on day 35 (both images shown), and images at P1 were acquired on day 51 and day 18 after passaging from P0 to P1. PCC, pheochromocytoma; P, passage.

Microscopically, organoids appeared as compact, roughly spherical structures with generally rounded but occasionally irregular contours and a dense cellular composition. Some organoids exhibited a more structured periphery, with concentric layering visible under bright-field microscopy. In certain cases, thin cellular projections extended outward, occasionally forming branch-like structures or apparent connections with nearby organoids or individual cells. No lumens were observed, and overall morphology remained consistent across passages (**Figure 3**).

**Figure 4:**
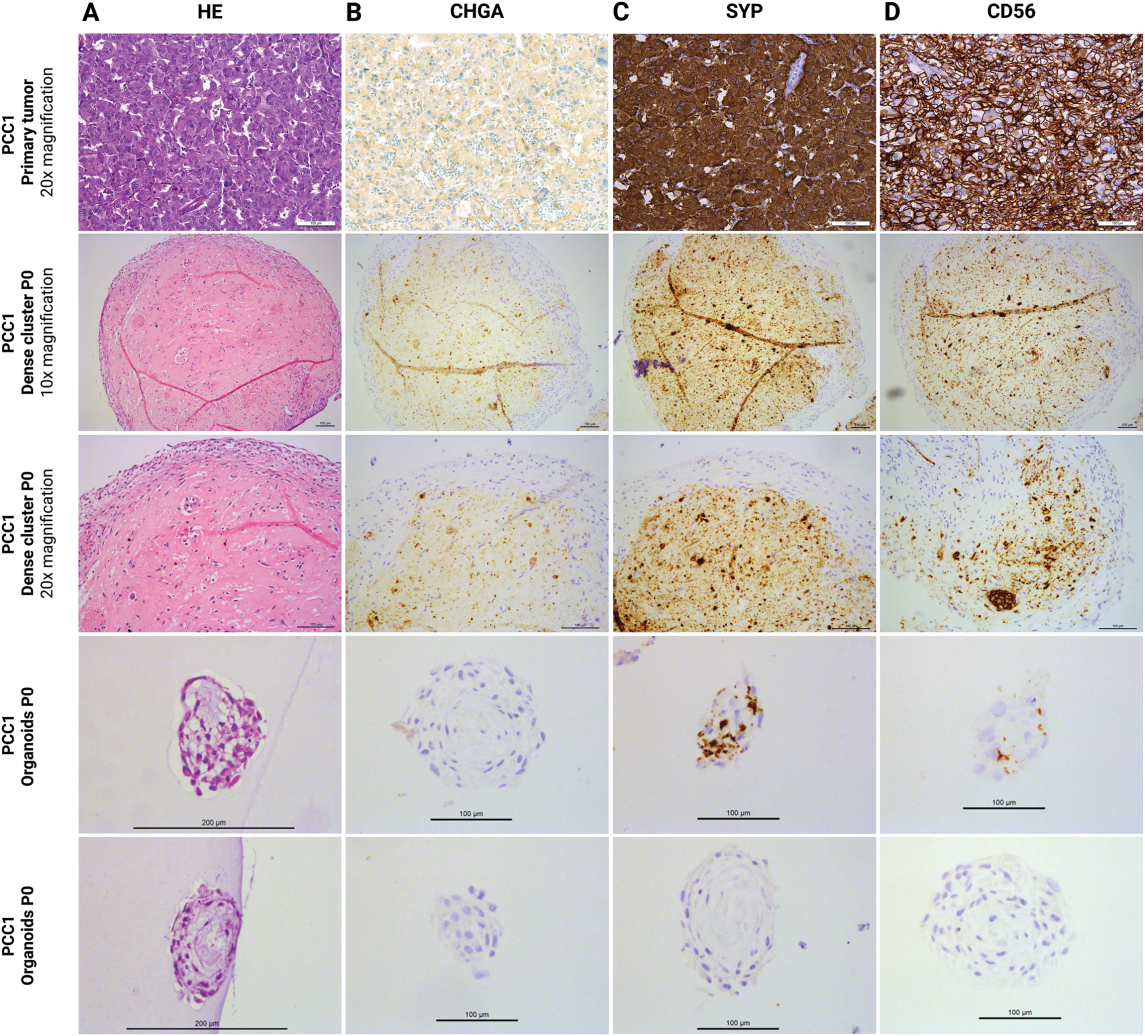
Histology **(A)** and IHC staining for differentiated chromaffin markers CHGA **(B)**, SYP **(C)**, and CD56 **(D)** in primary tumor tissue (PCC1) and its derived dense cellular clusters and organoids at the end of passage 0. IHC, immunohistochemistry; PCC, pheochromocytoma; P, passage; HE, hematoxylin and eosin; CHGA, chromogranin A; SYP, synaptophysin.

Histological analysis revealed that larger organoids frequently exhibited a central eosinophilic core with relatively few nuclei, surrounded by a peripheral layer of somewhat elongated, densely packed cells (**Figure 4A**). In contrast, smaller organoids lacked a central acellular region and displayed a more uniform cellular distribution. Dense cellular clusters showed interwoven bands of cell‐rich regions embedded in extracellular matrix, encircled by concentric bands of elongated peripheral cells (**Figure 4A**). Focal areas of cellular degeneration—characterized by cytoplasmic eosinophilia, nuclear pyknosis, and karyorrhexis—were observed in both large organoids and dense cellular clusters, indicative of apoptosis or necrosis (**Supplementary Figure 1**). Occasionally, macrophage‐like cells containing intracytoplasmic, hemosiderin‐like granules were noted in these regions.

### 3.4 Characterization of PCC organoids reveals the presence of both differentiated and progenitor cell populations

**Figure 5:**
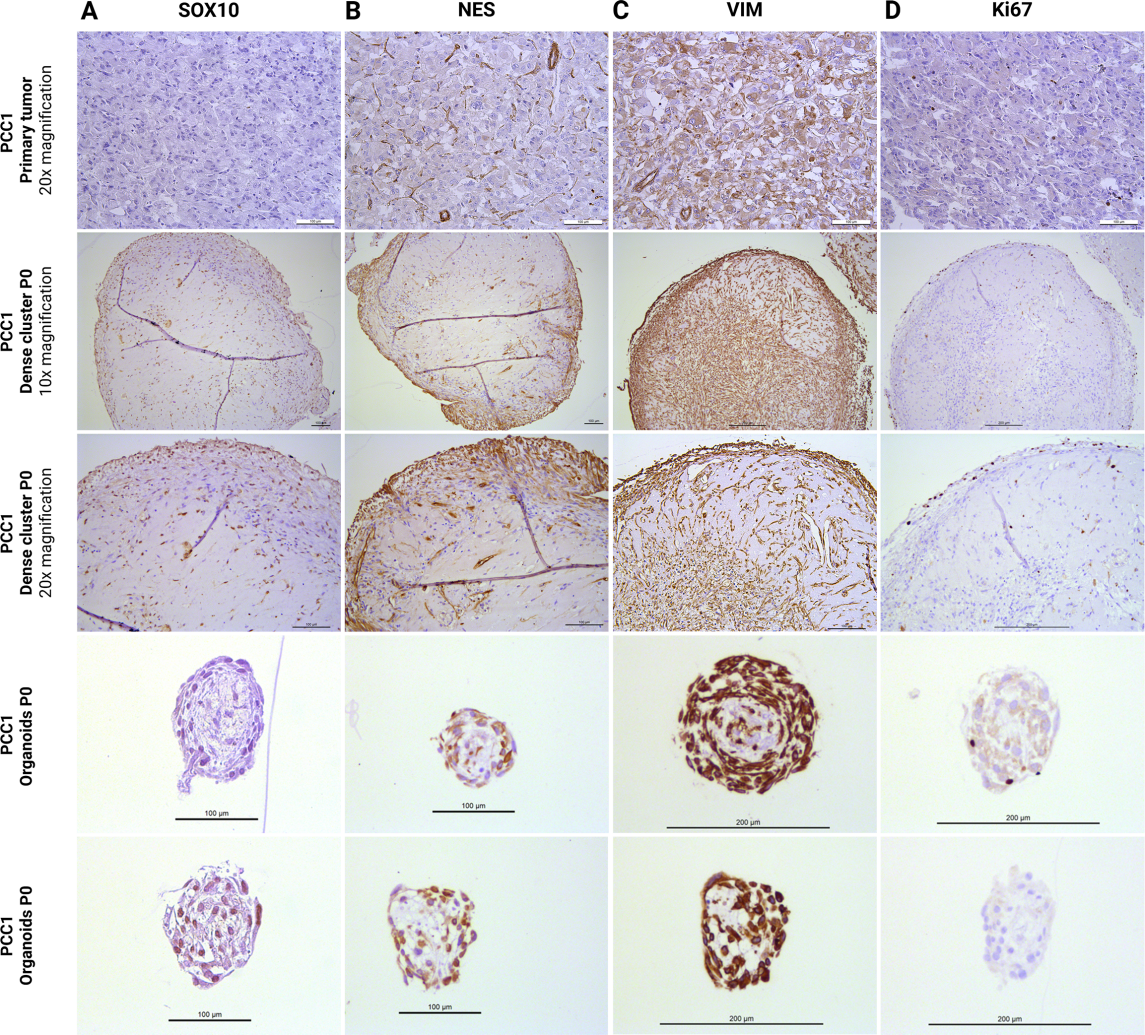
IHC staining for stem/progenitor markers SOX10 **(A)**, NES **(B)**, and VIM **(C)**, and the proliferation marker Ki67 **(D)** in primary tumor tissue (PCC1) and its derived dense cellular clusters and organoids at the end of passage 0. IHC, immunohistochemistry; PCC, pheochromocytoma; P, passage; SOX10, SRY-box transcription factor 10; VIM, vimentin; NES, nestin.

Immunohistochemical staining results for organoids and dense cellular clusters, compared to primary tumor tissue, are shown in **Figures 4**, **5**. These data are derived from PCC1, as only limited organoid material was available from PCC2, which was instead allocated to qPCR analysis, immunofluorescence (IF) analysis, and further passaging. Dense cellular clusters from PCC2 exhibited similar staining patterns to those from PCC1 (data not shown).

In PCC 1-derived organoids, markers indicative of differentiated chromaffin cells were inconsistently expressed. CHGA staining was not detected (**Figure 4B**), and SYP and CD56 were only observed in a subset of organoids, with others being entirely negative (**Figures 4C, D**). In dense cellular clusters, the staining for CHGA, SYP, and CD56 displayed a diffuse, pale granular pattern in central regions where extracellular matrix was more abundant, and was not clearly associated with cells (**Figures 4B–D**), suggesting nonspecific background staining. However, scattered individual cells or small cell groups within these clusters showed clear cytoplasmic staining, indicating specific expression of these differentiation markers (**Figures 4B–D**). These findings confirm the presence of chromaffin cells in both organoids and dense cellular clusters, albeit at lower levels than in primary tumor tissue.

Progenitor markers were more consistently expressed. SOX10 and NES staining was mainly localized to the outer cell layers of both organoids and dense cellular clusters, although positivity was also observed in more central regions (**Figures 5A, B**). SOX10 staining was heterogeneous among organoids, with some organoids showing no positive nuclei, while others displayed widespread nuclear positivity (**Figure 5A**). Vimentin staining was strong and uniformly distributed throughout both organoids and dense cellular clusters, and this pattern was consistent across all structures examined (**Figure 5C**). Ki67 staining revealed occasional proliferative cells, predominantly located in the outer layers of the organoids and dense cellular clusters (**Figure 5D**). Collectively, these findings indicate that PCC organoids and dense cellular clusters comprise a mixed population of differentiated chromaffin cells and stem/progenitor–like cells.

**Figure 6:**
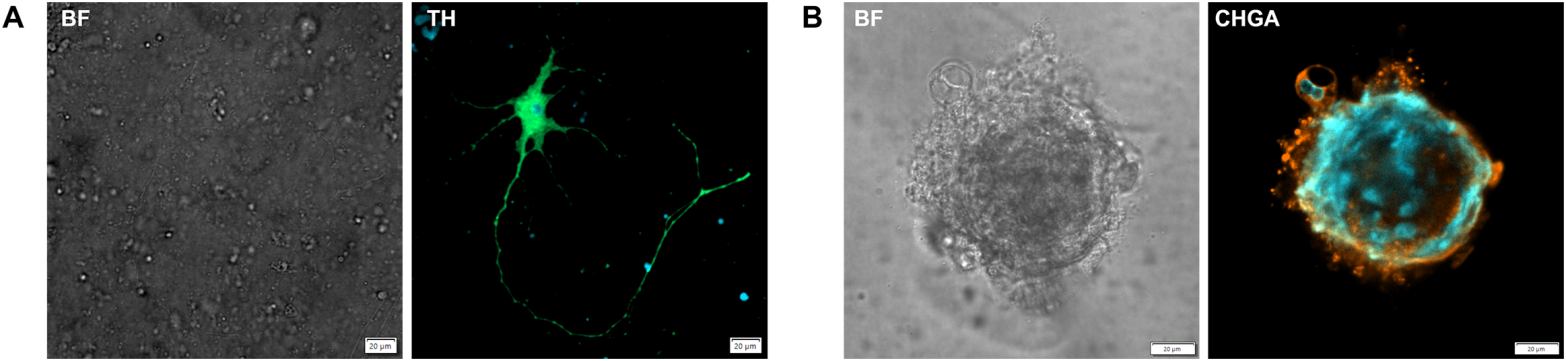
Immunofluorescence imaging of PCC organoid cultures showing staining for the differentiated chromaffin cell markers tyrosine hydroxylase (TH) and chromogranin A (CHGA). Markers are indicated at the top of each panel. Fluorescent signals are shown in green (Alexa Fluor 488) and orange (Alexa Fluor 568). Nuclei are counterstained with DAPI (blue). **(A)** A cell from PCC2 within the BME droplet at 16 days in culture (P0), displaying immunoreactivity for TH. The bright-field signal is limited due to imaging within the BME droplet. **(B)** An organoid from PCC2 at P1, 27 days in culture after passaging, with cells exhibiting immunoreactivity for CHGA. BF, bright field; TH, tyrosine hydroxylase; CHGA, chromogranin A; P, passage; PCC, pheochromoytoma.

**Figure 7:**
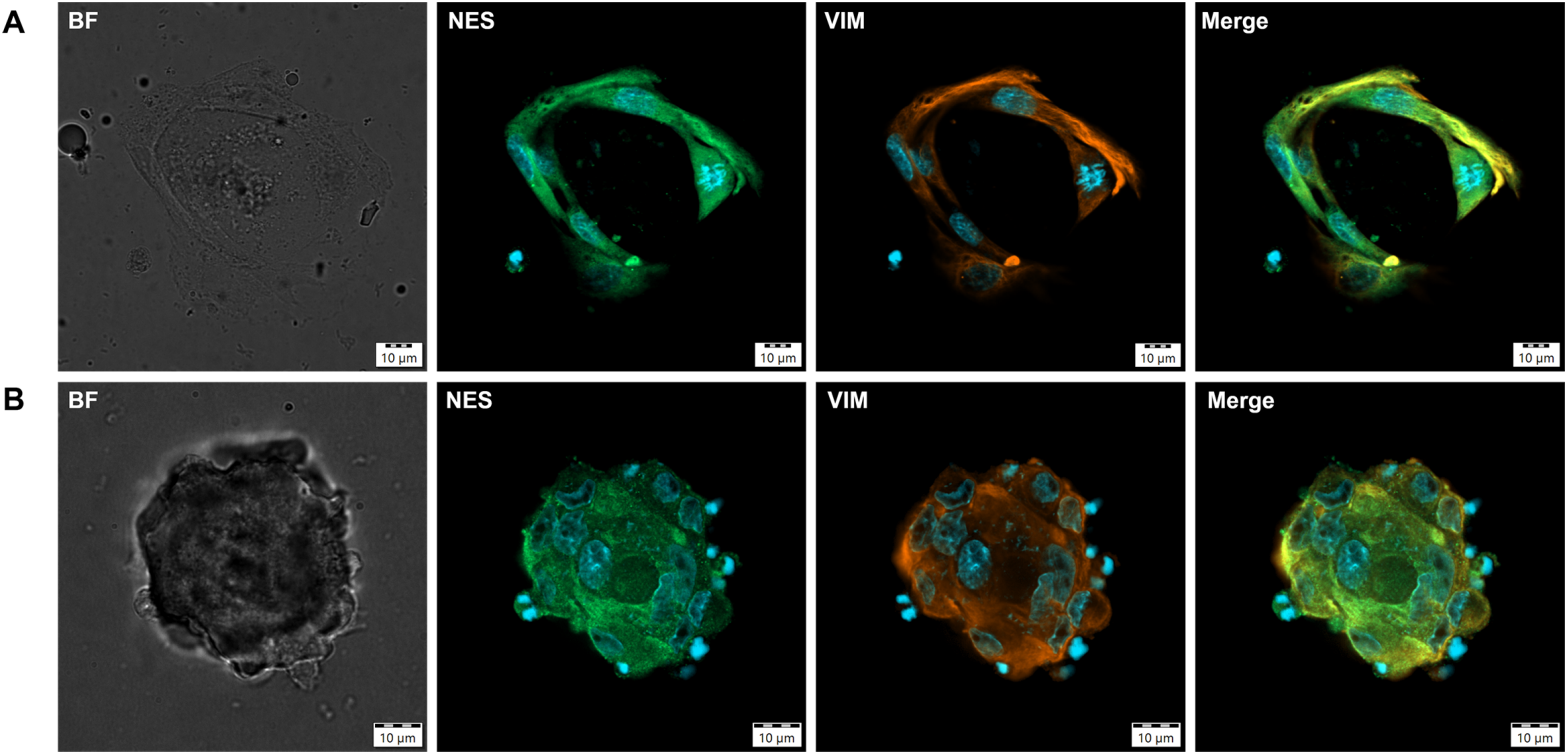
Immunofluorescence imaging of PCC organoid cultures showing staining for progenitor/stem cell markers nestin (NES) and vimentin (VIM). Markers are indicated at the top of each panel. Fluorescent signals are displayed as green (Alexa Fluor 488) and orange (Alexa Fluor 568). Nuclei are counterstained with DAPI (blue). **(A)** A cell cluster from PCC2 at 31 days in culture (P0), showing co-expression of NES and VIM. Note the mitotic figure in the cell on the right and the spindle-shaped morphology. **(B)** An organoid from PCC2 at 37 days in culture (P0), displaying immunoreactivity for NES and VIM. BF, bright field; NES, nestin; VIM, vimentin; PCC, pheochromoytoma; P, passage.

To further support these findings, IF staining was performed on organoid cultures from PCC2 at both P0 and P1. Despite the limited material available, these analyses confirmed the presence of both differentiated and progenitor markers. Specific fluorescence signal for the chromaffin differentiation markers TH and CHGA was observed within organoid cultures from PCC2 at P0 and P1 (**Figure 6**), while the stem/progenitor markers VIM and NES were detected in both PCC cell clusters outside the BME droplet (**Figure 7A**) and in organoids (**Figure 7B**) at P0. Fluorescence intensities ranged from 9- to 103-fold above background levels, as determined by normalization to negative controls, confirming specific marker expression. The detection of CHGA by IF, but not by IHC, likely reflects the higher sensitivity of fluorescence-based methods for low-level antigen expression. No consistent differences in IHC or IF staining patterns were observed between organoids cultured in WREFLD and WREFLD + IGF2 conditions.

**Figure 8:**
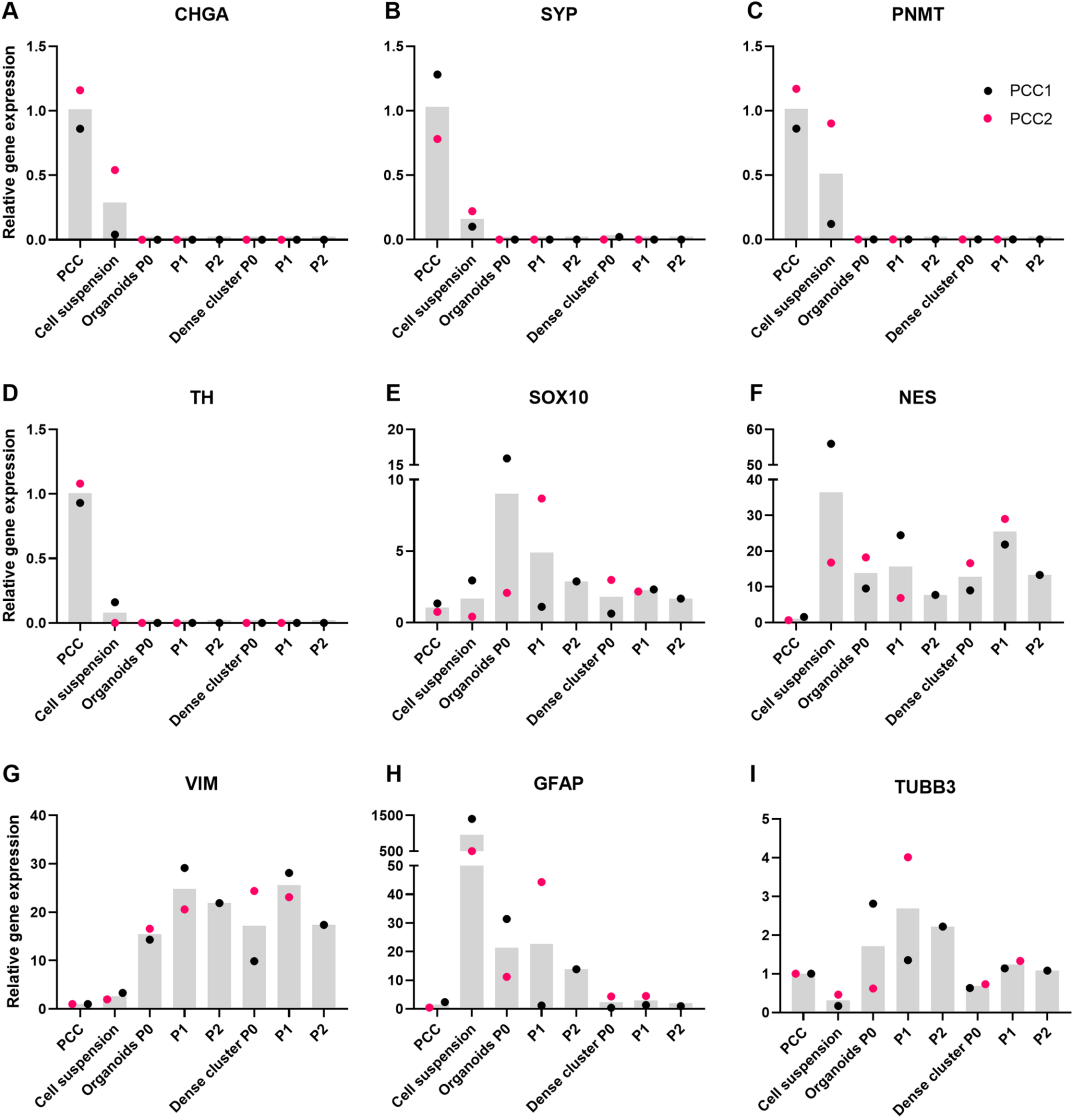
Relative gene expression levels of chromaffin cell markers *CHGA***(A)**, *SYP***(B)**, *PNMT***(C)**, and *TH***(D)**, adrenomedullary stem/progenitor markers *SOX10***(E)**, *NES***(F)**, *VIM***(G)**, and *GFAP***(H)**, and the neural marker *TUBB3***(I)** in PCC tissues, cell suspensions, and in organoids and dense cellular clusters at various passages (P0, P1, and P2). Bars represent median gene expression levels, while individual dots correspond to data from both PCCs. Statistical comparisons for each marker were performed using the Kruskal-Wallis test across all sample types (cell suspensions, organoids, and dense clusters at all available passages), with PCC tissue as the reference group. No statistically significant differences (*P* < 0.05) were found compared to PCC tissue for any of the markers; PCC, pheochromoytoma; P, passage.

qPCR analysis was performed on organoids and dense cellular clusters derived from both PCC1 (P0, P1, P2) and PCC2 (P0, P1). This analysis revealed a downward trend in the mRNA expression of adrenomedullary markers (*CHGA*, *SYP*, *PNMT*, and *TH*) in both organoids and dense cellular clusters compared to primary tumor tissue (**Figure 8**). In contrast, mRNA expression levels of stem/progenitor markers (*SOX10*, *NES*, *VIM*, and *GFAP*) were generally higher in organoids and dense clusters than in primary tumor tissue, although expression patterns varied across passages and between markers (**Figure 8**). Overall comparisons between groups using the Kruskal–Wallis test did not reveal statistically significant differences for any of the markers (*CHGA P* = 0.074; *SYP P* = 0.10; *PNMT P* = 0.081; *TH P* = 0.14; *SOX10 P* = 0.84; *NES P* = 0.24; *VIM P* = 0.15; *GFAP P* = 0.23). *TUBB3*, a neural marker, was detected in both organoids and dense clusters, with relatively stable expression across passages (**Figure 8**).

### 3.5 Differentiation of PCC organoids

To explore whether PCC organoids could be induced to differentiate, organoids were treated with Dex and/or PMA, with or without continued supplementation with WREFLD. This experiment was performed at P3 for PCC1, using 12 wells per differentiation condition and 24 wells for parallel expansion control conditions (WREFLD alone). In PCC2, differentiation was attempted at P2, but a fungal contamination compromised the culture, precluding meaningful interpretation of the results. Organoid formation was most robust in cultures maintained with WREFLD alone (24/24 wells) or WREFLD + Dex (12/12 wells, with robust growth in 9/12), followed by WREFLD + Dex + PMA (4/12 wells). In contrast, Dex alone yielded organoid formation in only 1/12 wells, and no organoid formation was observed in the Dex + PMA condition (0/12 wells).

**Figure 9:**
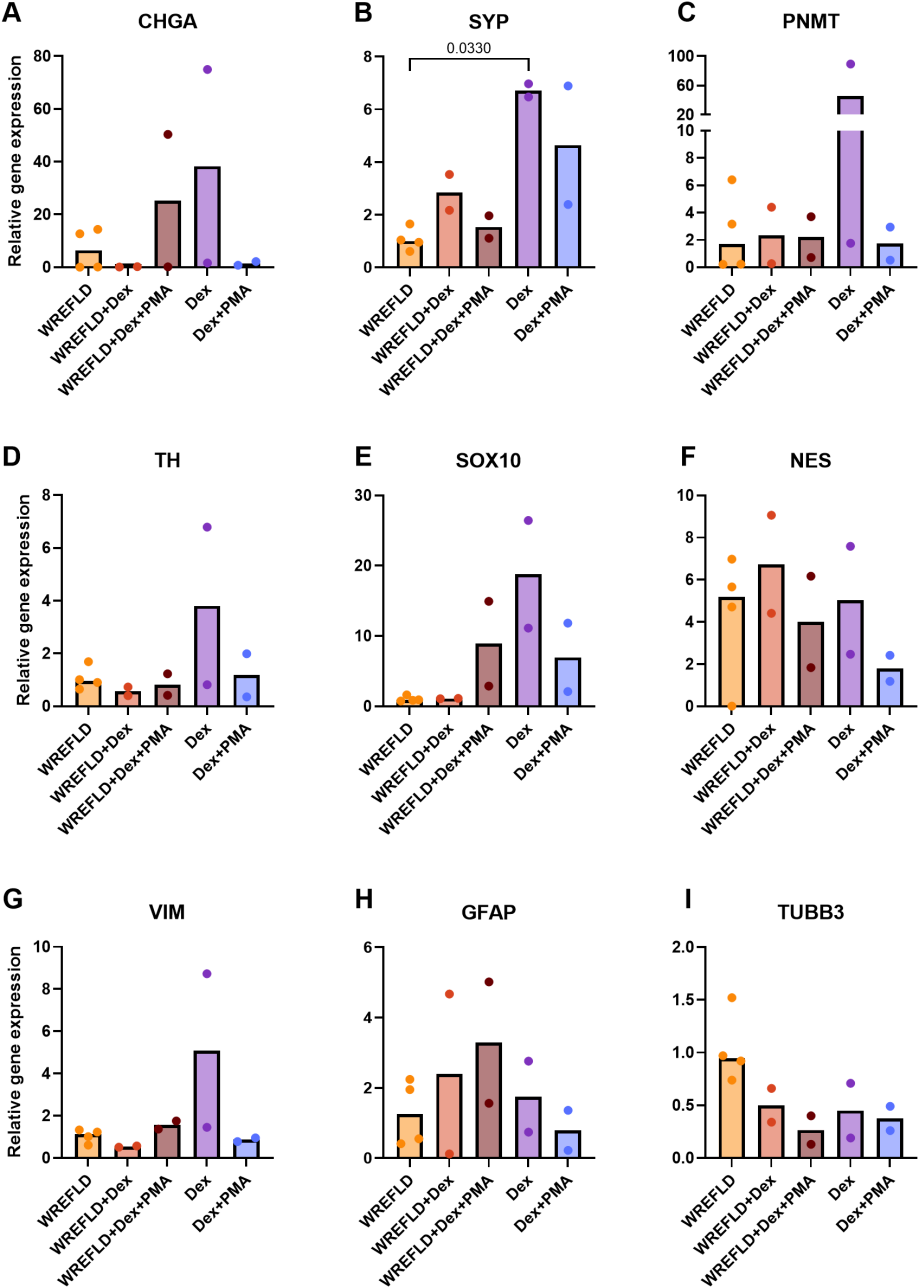
Relative gene expression levels of chromaffin cell markers *CHGA***(A)**, *SYP***(B)**, *PNMT***(C)**, and *TH***(D)**, adrenomedullary stem/progenitor markers *SOX10***(E)**, *NES***(F)**, *VIM***(G)**, and *GFAP***(H)**, and the neural marker *TUBB3***(I)** in PCC1 at P3 under different conditions. Each dot represents an individual technical replicate (n = 4 for WREFLD, n = 2 for all other conditions). Bars indicate median gene expression levels. All values are normalized to the WREFLD condition, which also served as the reference group for statistical comparisons (Kruskal–Wallis test with Dunn’s correction; *P* < 0.05). WREFLD, Wnt, R-spondin-3, EGF, FGF2, LIF, and DHEAS; Dex, dexamethasone; PMA, phorbol 12-myristate 13-acetate.

Due to the limited material availability, all organoids and dense cellular clusters were harvested for downstream qPCR analysis. Among the differentiation markers, *SYP* expression was significantly increased in the Dex condition compared to the WREFLD (expansion) condition (*P* = 0.030; **Figure 9**). Expression of other chromaffin markers (*CHGA*, *PNMT*, *TH*) appeared elevated in the same condition, but differences were not statistically significant. Stem/progenitor markers (*SOX10*, *NES*, *VIM*, *GFAP*) showed variable expression across conditions without a consistent pattern.

## 4 Discussion

This pilot study presents the first generation and characterization of patient-derived organoid cultures from human PCCs that were maintained over extended periods and serially passaged. Two organoid cultures were established and expanded across multiple passages, indicating the presence of proliferative potential. Immunohistochemical and immunofluorescence analyses confirmed the expression of stem/progenitor markers such as NES and SOX10, supporting the presence of a progenitor-like cell population within these organoids. In addition, organoid cultures expressed differentiation markers including CHGA, SYP, and CD56, and secreted detectable levels of metanephrines, reflecting functional characteristics of chromaffin cells. As cultures progressed, a decline in both differentiation marker expression and metanephrine production was observed, consistent with a gradual shift in cellular composition and functional state over time. Together, these findings support the feasibility of establishing human PCC organoid cultures as a proof-of-concept *in vitro* model and indicate that, with further optimization aimed at improving long-term stability, preserving functional differentiation, and enabling scalability, these cultures may provide a valuable platform for disease modeling and personalized drug screening in PCC.

One possible explanation for the observed decline in differentiation marker expression and metanephrine production is organoid dedifferentiation, a common phenomenon in organoid cultures whereby cells gradually revert to a less differentiated state during later passages (39). Alternatively, the reduced expression of differentiation markers may reflect selective depletion of differentiated chromaffin cells, resulting in relative enrichment of progenitor-like cells over time. If dedifferentiation were the primary mechanism, an associated increase in proliferative capacity might be expected. However, passage intervals remained relatively constant, and Ki67 staining did not indicate increased proliferation in later passages, suggesting that proliferation did not increase over time. This observation implies that additional mechanisms, such as oxygen-related cellular stress, may contribute not only to the loss of functional differentiation but also to the limited proliferative capacity despite the presence of progenitor-like cells. Histological analysis revealed focal areas of cellular degeneration in both organoids and dense cellular clusters, supporting the notion that viability may have been compromised in later stages of culture. As organoids increase in size, diffusion limitations are likely to create spatial gradients in oxygen and nutrient availability, leading to central hypoxia and nutrient scarcity that may impair differentiation and expansion (24, 40). At the same time, cultures were maintained in atmospheric oxygen conditions, which are hyperoxic relative to physiological adrenal tissue oxygen levels and may induce oxidative stress, further affecting cell viability and differentiation. Future studies should evaluate whether culturing PCC organoids under reduced oxygen tension improves growth kinetics and supports long-term maintenance of chromaffin features.

The relatively limited expansion observed may also be attributed to the intrinsic growth kinetics of PCC cells or culture conditions that may not have been optimal for sustained expansion. Organoids from PCC1 continued to form at P3, but the culture was terminated because available material was allocated to qPCR analysis, precluding further passaging. Nevertheless, growth across earlier passages was relatively slow, which may reflect the inherently low proliferative capacity of chromaffin cells—known to divide infrequently in the postnatal adrenal medulla (41)—and of PCCs, in which tumor doubling times can span several years or even decades (7). This is supported by Ki67 staining, which showed only sporadic positivity in the primary tumors of both PCC1 and PCC2, indicating that only a small subset of tumor cells was proliferating. This low proliferative index may contribute to the limited expansion observed *in vitro*. In addition, while the growth factor combination used in this study supported initial organoid formation, it may not have been optimal for maintaining robust and sustained proliferation. As tumor genotype may influence growth factor requirements, future studies incorporating tumors with diverse genotypes may reveal differential supplementation needs. Such insights, together with ongoing efforts to optimize culture conditions, could guide the refinement of growth factor combinations and the addition of specific niche factors to improve expansion and functional differentiation. In addition, co-culture systems with adrenocortical (tumor) cells might further enhance proliferation and help maintain differentiation (16, 42).

Beyond the organoids themselves, cultures frequently gave rise to large dense cellular clusters that displayed distinct morphological characteristics. Despite differences in size and structural organization, these dense clusters showed comparable marker expression profiles as the organoids, based on IHC and qPCR, supporting the presence of both differentiated and progenitor-like cell populations. They formed within the BME droplet and were characterized by abundant extracellular matrix and numerous cells with a mesenchymal-like morphology, displaying contractile and motile behavior during culture, which gradually condensed into compact structures. Interestingly, their morphological features resemble the fetal “adrenal organoids” described by Poli et al. (2019) (43), which also featured nestin-positive, motile mesenchymal cells embedded in matrix-rich regions. Whether these dense clusters represent a supportive niche or a distinct growth modality remains to be determined.

Other efforts to establish human PPGL organoids have primarily focused on short-term cultures designed for rapid assessment of therapeutic responses. In a conference abstract, Calucho et al. (2023) (44) described PPGL organoids generated in a mini-ring format optimized for short-term drug screening without prior *in vitro* expansion, and more recently, Erali et al. (2025) (27) reported patient-derived PPGL organoids to assess drug-induced effects on cell viability. According to commonly used definitions, organoids are three-dimensional structures derived from stem, progenitor, and/or differentiated cells that self-organize to recapitulate aspects of native tissue architecture and function *in vitro* (45). Beyond three-dimensional growth alone, sustained maintenance with serial passaging allows assessment of organoid behavior over time. In the present study, human PCC-derived organoids were maintained over extended periods and serially passaged, enabling longitudinal characterization of cellular identity and functional changes, thereby complementing prior PPGL culture systems primarily designed for short-term drug response assessment.

To assess the effects of the applied differentiation-inducing conditions on PCC organoid cultures, qPCR analysis was performed following exposure to differentiation stimuli (19, 46, 47). These analyses showed a trend toward upregulation of differentiation markers such as *CHGA*, *SYP*, *PNMT*, and *TH* in the Dex condition, with *SYP* reaching statistical significance. However, this condition did not support consistent organoid formation, suggesting that the observed gene expression changes primarily reflect responses of cells present in the culture rather than within structured organoids. These findings raise the possibility that the timing of exposure to differentiation stimuli may influence outcomes; allowing organoids to first establish under expansion conditions before applying differentiation cues might support more effective maturation. The lack of clear upregulation in the other conditions may reflect insufficient differentiation under the applied conditions or indicate that only a subset of cells responded to the stimuli—an effect that may not be detectable through bulk qPCR analysis, which cannot capture expression heterogeneity at the single-cell level. Single-cell RNA sequencing would be the preferred strategy in future studies to identify and quantify the different cell populations present and to track shifts in chromaffin differentiation states over time, particularly when combined with more refined differentiation conditions, thereby providing deeper insight into the differentiation potential and cellular dynamics of PCC organoids (48). For future drug screening applications, maintaining differentiated features may be important to ensure that certain drug responses observed *in vitro* accurately reflect clinical responses (24); however, organoid models may also retain value by capturing tumor-intrinsic properties (e.g., genetic drivers or pathway dependencies), even if cultures shift toward less differentiated states.

Expression of SOX10, NES, and VIM has been described in adrenomedullary progenitors with differentiation potential toward chromaffin, glial, and neuronal lineages (19, 20, 34), and may indicate the presence of sustentacular-derived progenitor cells in our organoid cultures. This is further supported by the observation that some cells exhibited a mesenchymal morphology not typical of differentiated chromaffin cells, but more compatible with a sustentacular phenotype (6, 8). Although such morphology may also suggest overgrowth by fibroblasts or myofibroblasts—previously observed in long-term PPGL cultures (49)—the expression of SOX10 and NES argues against a purely stromal origin. Nevertheless, the precise identity of these cells would require lineage tracing or single-cell transcriptomic profiling, which will be an important focus of future studies. Our findings parallel several observations reported by Bayley et al. (2022) (49) in their study on long-term 2D culture of human PPGLs, including the progressive decline in chromaffin differentiation markers over time, the emergence of mesenchymal-appearing cells, and the formation of dense cellular clusters somewhat resembling the cell agglomerations described in their cultures. Combining their insights (e.g., addition of lactate to promote chromaffin cell survival) with unexplored strategies such as BMP4 and NGF exposure during differentiation (50) may enhance the survival and maturation of chromaffin cells and further improve the physiological relevance of PCC organoids.

This study has several limitations. First, only two PCCs were included, and although organoid expansion was achieved, culture duration was ultimately limited either by allocation of material for downstream analyses or by contamination. Longer-term expansion and more extensive differentiation experiments would therefore be desirable in future studies. Second, while differentiation was assessed at the transcript level by qPCR, complementary analyses such as hormone secretion assays and protein expression studies under differentiation conditions were not performed. Third, transcriptomic and genomic profiling was not conducted, limiting deeper molecular characterization of the organoid cultures. Future efforts should focus on optimizing culture conditions to promote proliferation and differentiation, establishing organoid lines from genetically diverse tumors, and performing molecular profiling (e.g., single-cell RNA sequencing and whole genome sequencing) of organoids to further confirm their resemblance to the primary tumors (51).

Despite these limitations, the establishment of patient-derived PCC organoids that could be expanded and serially passaged represents an important step forward. Organoid cultures offer the potential for expansion, with cryopreservation and biobanking representing important future steps, and may enable high-throughput drug testing (24, 25). In this study, both organoid cultures were derived from PCCs without known susceptibility gene mutations, underscoring the importance of including genetically diverse tumors. A genetically annotated PCC organoid biobank could facilitate molecular subtyping and support the development of cluster- or even mutation-specific therapies (26).

Material availability remains a key hurdle in rare tumors like PCC. In parallel to our work on human PCC organoids, our group has developed canine PCC and adrenomedullary organoid cultures. PCCs occur naturally in dogs, are more prevalent, and share clinical, biochemical, histological, and genetic features with human PCCs, making them a promising translational model (52–54). We recently established long-term canine PCC and adrenomedullary organoid cultures, including from frozen primary tumor material and from cryopreserved organoids (28). These canine organoid models not only offer opportunities to refine culture protocols and biobanking strategies for human PCC organoids, but also provide a complementary platform for translational research and therapeutic development. Together, these approaches may accelerate drug discovery and improve access to functional models of this rare tumor type.

In conclusion, this pilot study demonstrates the feasibility of generating patient-derived organoids from human PCC tissue that can be maintained in long-term culture and serially passaged. In early stages, these organoids retain key phenotypic and functional traits of their original tumors, but further optimization is needed to enhance their long-term proliferation and differentiation potential. Achieving stable growth and differentiation would be required for PCC organoids to more closely reflect *in vivo* tumor biology and to serve as a translational platform for studying PCC pathogenesis and supporting the development and testing of novel therapies, ultimately advancing personalized medicine for PCC patients.

## Data Availability

The data supporting the findings of this study are included in the article and its **Supplementary Material**. Additional data are available from the corresponding author upon reasonable request.

## Glossary

BME: basement membrane extract
CHGA: chromogranin A
DAPI: 4’,6-diamidino-2-phenylindole
Dex: dexamethasone
DHEAS: dehydroepiandrosterone sulfate
DMEM: Dulbecco’s Modified Eagle Medium
EGF: epidermal growth factor
EM: expansion medium
FCS: fetal calf serum
FGF2: fibroblast growth factor 2
GFAP: glial fibrillary acidic protein
GUSB: glucuronidase beta
HPRT1: hypoxanthine phosphoribosyltransferase
IF: immunofluorescence
IHC: immunohistochemistry
LC-MS/MS: liquid chromatography-tandem mass spectrometry
LIF: leukemia inhibitory factor
NES: nestin
P: passage
PCC: pheochromocytoma
PFA: paraformaldehyde
PGL: paraganglioma
PMA: phorbol 12-myristate 13-acetate
PNMT: phenylethanolamine N-methyltransferase
PPGL: pheochromocytoma and paraganglioma
P/S: penicillin/streptomycin
qPCR: quantitative real-time reverse transcription polymerase chain reaction
SOX10: SRY-box transcription factor 10
SYP: synaptophysin
TH: tyrosine hydroxylase
TUBB3: tubulin beta 3 class III
VIM: vimentin
WREFLD: combination of growth factors Wnt, R-spondin-3, EGF, FGF2, LIF, and DHEAS
YWHAZ: tyrosine 3-monooxygenase/tryptophan 5-monooxygenase activation protein zeta.
