# Supplementary material for: Long-term culture of patient-derived pheochromocytoma organoids
Source: Front Endocrinol (Lausanne). 2026 Mar 18;17:1781556. doi: 10.3389/fendo.2026.1781556 (PMC13038448; doi:10.3389/fendo.2026.1781556)
Supplement: Supplementary file 1 [file DataSheet1.docx]

Supplementary Material

# Supplementary File 1. Extended Materials and Methods

**Tumor dissociation and single-cell preparation**Tumor tissue was processed immediately upon arrival. Tissue fragments were cut into approximately 1 mm^3^ pieces and enzymatically digested using 1 mg/mL collagenase 1A (Sigma-Aldrich, Merck KGaA, Darmstadt, Germany) and 2500 units/mL DNAse I (Gibco) in Advanced DMEM/F-12 (Gibco), supplemented with 1% (v/v) P/S, 1% (v/v) GlutaMAX (Gibco), and 10 mM 1% (v/v) HEPES (Gibco). Digestion was performed in a shaking water bath at 37°C, using 10 mL of digestion medium per 1 cm³ of tissue. Tissue fragments were vortexed every 2 min and mechanically dissociated by pipetting at 5-min intervals, using progressively smaller pipette tips. After allowing the undigested fragments to settle, the supernatant containing dissociated cells was collected. Fresh enzyme-containing medium was added to the remaining fragments every 10 min, for up to three digestion cycles. Following enzymatic and mechanical dissociation, the supernatant was pooled and cells were washed in cold DMEM/F-12 supplemented with 10% (v/v) FCS and 1% (v/v) P/S to inactivate collagenase, followed by centrifugation at 240 *g* for 5 min at 4°C. If the resulting cell pellet appeared red, indicating the presence of red blood cells (RBC), RBC lysis was performed by adding 1–2 mL of 1x RBC lysis buffer (Roche, Mannheim, Germany) and incubating for 5 min at room temperature (RT). The reaction was stopped by adding twice the volume of DMEM/F-12 with 10% (v/v) FCS and 1% (v/v) P/S, followed by centrifugation. RBC lysis was repeated as necessary based on the pellet’s color. Finally, cells were counted using an automated cell counter (Bio-Rad, Hercules, CA, USA) and resuspended in Advanced DMEM/F-12 supplemented with 1% (v/v) P/S, 1% (v/v) GlutaMAX, 10 mM 1% (v/v) HEPES, and 10 µM Y-27632 dihydrochloride (AbMole BioScience, Houston, USA).

**Organoid culture conditions and medium composition**Single-cell suspensions were combined with cold Cultrex® basement membrane extract (BME; R&D Systems, Minneapolis, MN, USA) at a 1:3 volume ratio and dispensed as 15 μL droplets onto pre-warmed 48-well plates (Greiner Bio-One, Frickenhausen, Germany). After a 30-min gelation period at 37°C, pre-warmed expansion medium (EM) was added to the solidified droplets. Cultures were maintained at 37°C in a humidified incubator with 5% CO₂. The EM, previously optimized for canine adrenal tissue, consisted of Advanced DMEM/F-12 (Gibco) supplemented with 1% (v/v) P/S, 1% (v/v) GlutaMAX (Gibco), 10 mM 1% (v/v) HEPES (Gibco), 2% (v/v) B27 supplement without vitamin A (Gibco), 1% (v/v) N2 supplement (Gibco), 1 mM N-acetylcysteine (NAC; Sigma-Aldrich), and 2 mM calcium gluconate (Sigma-Aldrich). Upon establishment of new organoid cultures, 10 µM Y-27632 dihydrochloride (AbMole BioScience, Houston, USA) and primocin (InvivoGen, San Diego, CA, USA) were added once. To promote organoid growth, the EM was supplemented with a growth factor combination referred to as WREFLD, containing 0.25 nM Wnt surrogate (ImmunoPrecise Antibodies, Utrecht, The Netherlands), 2% (v/v) R-spondin-3 (ImmunoPrecise Antibodies), 20 ng/mL epidermal growth factor (EGF; Peprotech, Cranbury, NJ, USA), 20 ng/mL fibroblast growth factor 2 (FGF2; Peprotech), 20 ng/mL recombinant human leukemia inhibitory factor (LIF; Peprotech), and 2 μM dehydroepiandrosterone sulfate (DHEAS; Cerilliant, Texas, USA). Half of the culture wells were additionally supplemented with 100 ng/mL insulin-like growth factor 2 (IGF2; Fujifilm, Irvine Scientific, Santa Ana, CA, USA), while the other half contained only WREFLD. As a negative control, a separate plate received only EM without added growth factors. The medium, including WREFLD (with or without IGF2), was refreshed 2-3 times per week by removing half of the supernatant and replacing it with fresh medium.

Organoid growth and morphology were monitored every 3–4 days using an Olympus CKX53 inverted microscope (Olympus, Tokyo, Japan). Brightfield images were acquired with a Leica Flexacam C3 camera and Leica Application Suite X software (Version 5.2.1.27831; Leica Microsystems, Wetzlar, Germany).

**Organoid passaging protocol**To evaluate culture maintenance and passaging over time, we applied a passaging protocol involving the separate dissociation of organoids and dense cellular clusters, after which the dissociated cells were combined and replated. Culture medium was collected before dissociation to preserve secreted factors for use in subsequent passages. Dense cellular clusters were harvested into 15 mL tubes on ice, centrifuged at 200 *g* for 4 min at 4°C, and the pellet was treated with accutase (200 µL per well) for 10 min at 37°C with gentle vortexing every 2 min. Dissociation was facilitated by pipetting with progressively smaller tips until all fragments could pass through an uncut 200 µL pipette tip. If dissociation was incomplete, collagenase 1A (0.5 mg/mL) was added, followed by incubation in a shaking water bath at 37°C for up to 20 min with repeated pipetting using progressively smaller pipette tips until complete dissociation. Cells were then washed in cold DMEM/F-12 supplemented with 10% (v/v) FCS and 1% (v/v) P/S, centrifuged, and stored at 4°C. Organoids were collected by washing wells twice with cold DMEM/F-12 supplemented with 10% (v/v) FCS and 1% (v/v) P/S, followed by gentle scraping to detach organoids and centrifugation (200 g, 4 min, 4°C). Pellets were resuspended in accutase (100 µL per well) and incubated at 37°C for 10 min in a microshaker set at 200 rpm. If organoids remained attached to the well bottom, additional accutase (100 µL per well) was added directly to the wells, followed by a further 10-min incubation at 37°C. After incubation, cells were washed and centrifuged as described above. Dissociated cells from dense clusters and organoids were combined and resuspended in Advanced DMEM/F-12 supplemented with 1% (v/v) P/S, 1% (v/v) GlutaMAX, 10 mM 1% (v/v) HEPES, and 10 µM Y-27632 dihydrochloride. Cells were replated at a ratio of 1:1.5 to 1:2, corresponding to seeding 1.5 to 2 new wells per original well. The cell suspension was mixed with cold Cultrex® BME at a 1:3 volume ratio, and 15 µL droplets were plated in 48-well plates. Pre-warmed EM containing 40% (v/v) conditioned medium from the previous passage and 60% (v/v) fresh EM with WREFLD and 20 µM Y-27632 was added. IGF2 was added to half of the culture wells.

**Histology and immunohistochemistry protocol**Organoids and dense cellular clusters were fixed in 4% (v/v) buffered paraformaldehyde (PFA), embedded in agarose, dehydrated in 70% (v/v) ethanol, and paraffin-embedded, as previously described (1). Primary tumors were fixed in 4% (v/v) PFA and directly paraffin-embedded. Sections (4 μm) were stained with hematoxylin and eosin or subjected to IHC. For CHGA, SYP, and CD56, IHC was performed using the fully automated staining platform Omnis (DAKO Agilent Technologies, Glostrup, Denmark) with the EnVision FLEX detection system (DAKO Agilent Technologies), according to the manufacturer’s standard protocols. For other markers (VIM, NES, SOX10, Ki67), manual IHC was conducted following the same protocol as previously described (1), with antibody-specific details provided in Supplementary Table 1. For quality control, positive control tissues were included for each antibody, while negative controls were prepared by omitting the primary antibody.

**Immunofluorescence staining protocol**For immunofluorescence (IF) analysis, cell suspensions mixed with BME were plated as 30 μL droplets onto pre-warmed 4-compartment CellView™ cell culture dishes (Greiner Bio One), after which expansion medium supplemented with WREFLD was added. Organoids were fixed using 3% (v/v) PFA (Electron Microscopy Sciences, Hatfield, PA, USA) in 0.2M sodium phosphate buffer supplemented with 0.1% (v/v) glutaraldehyde (Sigma-Aldrich) and phosphate buffered saline (PBS) for 30 min at RT. After fixation, organoids were immersed in 75% (v/v) ethanol and stored at 4°C for up to one month before further processing. For staining, organoids were washed four times with PBS for 10-15 min at RT on a rocking platform. PFA was quenched by incubating the organoids in PBS containing 0.1% (w/v) sodium borohydride (Sigma-Aldrich) for 10 min at RT on a rocking platform. Non-specific antibody binding was blocked using 5% (v/v) NGS and 0.1% (w/v) saponin in PBS for 1 h at RT. Organoids were then incubated with primary antibodies diluted in PBS containing 1% (w/v) BSA and 0.1% (w/v) saponin for at least 24 h at 4°C. Following incubation, organoids were washed four times with PBS containing 0.1% (w/v) saponin for 10–15 min at RT on a rocking platform. Next, secondary antibodies were applied and incubated either for 6 h at RT or overnight at 4°C under the same conditions. Organoids were washed again and subjected to nuclear staining with 4’,6-diamidino-2-phenylindole (DAPI, 1:400; R&D, Minneapolis, MN, USA) in PBS for 10–15 min at RT in the dark on a rocking platform. Finally, organoids were rinsed with autoclaved demineralized water and mounted with VectaMount® Permanent Mounting Medium (H5000; Vector Laboratories, Newark, CA, USA). Supplementary Table 2 lists the details of the primary and secondary antibodies used in this study. Before use, all antibodies were centrifuged at 13,200 *g* at 4°C for 30 min.

**Image acquisition**Images were acquired using an Olympus SpinSR10 spinning disk confocal microscope (Olympus) equipped with ORCA fusion sCMOS cameras (Hamamatsu Photonics, Hamamatsu, Japan) and a 30x silicone oil immersion objective (UPLXAPO S 30x, NA1.05; Olympus). Typically, organoids were imaged as single slices at the mid-plane of their height. Sequential laser triggering (Coherent-OBIS, Saxonburg, PA, USA) was applied using the following settings: 405 nm (50 mW, set at 60%), 488 nm (100 mW, set at 40%), 561 nm (100 mW, set at 50%), and 640 nm (100 mW, set at 50%). Laser excitation was synchronized with fluorescence emission filters (AHF Analysentechnik GmbH, Tübingen, Germany) via cellSens Dimensions software (version 4.2, Olympus). Fluorescence detection used the following filter sets: B477/60 for DAPI, B525/50 for Alexa Fluor™ 488, B607/36 for Alexa Fluor™ 568, and B685/40 for Alexa Fluor™ 647, with a 100 ms exposure time for each channel. A quadband dichroic mirror (D405/488/561/640 nm) was utilized. When indicated, Z stacks were acquired covering the entire organoid volume in 0.41 µm steps. Resulting images, including side views and maximum intensity projections, were generated using cellSens software. A 50 µm pinhole SoRa spinning disk was positioned in the optical path, without the 3.2 magnifying lens, to improve detection sensitivity. All technical settings were standardized across experiments to ensure consistent image acquisition.

**RNA isolation and quantitative real-time RT-PCR**Total RNA was isolated from PCC tumor tissue, cell suspensions, organoids, and dense cellular clusters at different passages using the RNeasy Mini Kit (Qiagen, Hilden, Germany), according to the manufacturer’s instructions. All isolations were performed with on-column DNase digestion using the RNase-Free DNase Set (Qiagen) to eliminate genomic DNA contamination. For samples cultured under WREFLD and WREFLD + IGF2 conditions, RNA was combined to ensure sufficient material for analysis. Additional RNA was collected from PCC1 after the differentiation experiment at P3. RNA concentrations were measured using a NanoDrop ND-1000 Spectrophotometer (Thermo Fisher Scientific), and purity was assessed by A260/280 and A260/230 ratios. cDNA was synthesized from 300 ng total RNA using SuperScript™ II Reverse Transcriptase (Thermo Fisher Scientific) and random primers. Quantitative real-time RT-PCR (qPCR) analyses were performed, using SYBR Green Mastermix (Bio-Rad) and a CFX96 qPCR machine (Bio-Rad), to assess the expression of adrenomedullary markers (*CHGA*, *SYP*, *TH*, *PNMT*), stem/progenitor markers (*NES*, *VIM*, *SOX10*, *GFAP*), and neural markers (*TUBB3*). Messenger RNA and genomic sequences for all target genes were obtained from the NCBI GenBank database. Primers (Supplementary Table 3) were designed using PerlPrimer version 1.1.21 (2), following the Bio-Rad iCycler guidelines, and checked for secondary structures using the mFold web server (<http://www.unafold.org/mfold/applications/dna-folding-form.php>) (3). Primers were ordered from Eurogentec (Liège, Seraing, Belgium) and validated for specificity and efficiency. Six candidate reference genes (glyceraldehyde-3-phosphate dehydrogenase [*GAPDH*], tyrosine 3-monooxygenase/tryptophan 5-monooxygenase activation protein zeta [*YWHAZ*], beta-2-microglobulin [*B2M*], glucuronidase beta [*GUSB*], hypoxanthine-guanine phosphoribosyltransferase 1 [*HPRT1*], and eukaryotic translation elongation factor 1 alpha 1 [*EEF1A1*]) were evaluated for stability using the geNorm algorithm (4). *YWHAZ*, *GUSB*, and *HPRT1* were selected based on pairwise variation and expression stability across sample types. Relative gene expression levels were calculated using the 2^–ΔΔCt method (5).

**References**

1. van den Berg MF, Timmermans-Sprang EPM, Viets FC, van den Berg L, Danawar F, van Wolferen ME, Kooistra HS, Grinwis GCM, de Jong WHA, van Faassen M, et al. Canine Adrenomedullary and Pheochromocytoma Organoids: A Novel In Vitro Model. *Endocrinology* (2025) 166: doi: 10.1210/endocr/bqaf114

2. Marshall OJ. PerlPrimer: cross-platform, graphical primer design for standard, bisulphite and real-time PCR. *Bioinformatics* (2004) 20:2471–2472. doi: 10.1093/bioinformatics/bth254

3. Zuker M. Mfold web server for nucleic acid folding and hybridization prediction. *Nucleic Acids Res* (2003) 31:3406–3415. doi: 10.1093/nar/gkg595

4. Vandesompele J, De Preter K, Pattyn ilip, Poppe B, Van Roy N, De Paepe A, Speleman rank. Accurate normalization of real-time quantitative RT-PCR data by geometric averaging of multiple internal control genes. (2002). http://genomebiology.com/2002/3/7/research/0034.1Correspondence:.rankSpeleman.

5. Livak KJ, Schmittgen TD. Analysis of Relative Gene Expression Data Using Real-Time Quantitative PCR and the 2−ΔΔCT Method. *Methods* (2001) 25:402–408. doi: https://doi.org/10.1006/meth.2001.1262

# Supplementary Tables

**Supplementary Table 1**. List of antibodies used in IHC analyses.

| Antibody | Host | Research Resource Identifier | Antigen retrieval | Dilution |
| --- | --- | --- | --- | --- |
| CHGA | Mouse | Thermo Fisher Scientific Cat# MA5-13096 [RRID:AB_10987033](https://scicrunch.org/resolver/RRID:AB_10987033) | Tris-EDTA, 97°C, 30 min | 1:800 |
| SYP | Mouse | Agilent Cat# GA660, [RRID:AB_3698021](https://scicrunch.org/resolver/AB_3698021) | Tris-EDTA, 97°C, 30 min | N/A |
| CD56 | Mouse | Agilent Cat# M730429-2, [RRID:AB_2750583](https://scicrunch.org/resolver/AB_2750583) | Tris-EDTA, 97°C, 30 min | N/A |
| SOX10 | Mouse | Santa Cruz Biotechnology Cat# sc-365692, [RRID:AB_10844002](https://scicrunch.org/resolver/AB_10844002) | Tris-EDTA, 98°C, 30 min | 1:500 |
| NES | Rabbit | Thermo Fisher Scientific Cat# PA5-11887, [RRID:AB_2148923](https://scicrunch.org/resolver/AB_2148923) | Citrate, 98°C, 30 min | 1:500 |
| VIM | Mouse | BioGenex Cat# AM074GP, [RRID:AB_3101770](https://scicrunch.org/resolver/AB_3101770) | Citrate, 98°C, 60 min | 1:500 |
| Ki67 | Mouse | Agilent Cat# M7240, [RRID:AB_2142367](https://scicrunch.org/resolver/AB_2142367) | Tris-EDTA, 98°C, 30 min | 1:200 |
| Anti-mouse IgG | Goat | ImmunoLogic Cat# DPVM110HRP, [RRID:AB_2915957](https://scicrunch.org/resolver/AB_2915957) |  |  |
| Anti-rabbit IgG | Goat | ImmunoLogic Cat# DPVR110HRP, [RRID:AB_2915958](https://scicrunch.org/resolver/AB_2915958) |  |  |

IHC, immunohistochemistry; CHGA, chromogranin A; SYP, synaptophysin; SOX10, SRY-box transcription factor 10; NES, nestin; VIM, vimentin; N/A, not applicable.

**Supplementary Table 2.** List of antibodies used in IF analyses.

| Antibody | Host | Research Resource Identifier | Dilution |
| --- | --- | --- | --- |
| CHGA | Mouse | Thermo Fisher Scientific Cat# MA5-13096, [RRID:AB_10987033](https://scicrunch.org/resolver/AB_10987033) | 1:100 |
| TH | Rabbit | Novus Cat# NB300-109, [RRID:AB_10077691](https://scicrunch.org/resolver/AB_10077691) | 1:1000 |
| NES | Rabbit | Thermo Fisher Scientific Cat# PA5-11887, [RRID:AB_2148923](https://scicrunch.org/resolver/AB_2148923) | 1:300 |
| VIM | Mouse | BioGenex Cat# AM074GP, [RRID:AB_3101770](https://scicrunch.org/resolver/AB_3101770) | 1:80 |
| Anti-rabbit IgG  Alexa Fluor™ 488 | Goat | Thermo Fisher Scientific Cat# A-11008, [RRID:AB_143165](https://scicrunch.org/resolver/AB_143165) | 1:1000 |
| Anti-mouse IgG  Alexa Fluor™ 568 | Goat | Thermo Fisher Scientific Cat# A-11004, [RRID:AB_2534072](https://scicrunch.org/resolver/AB_2534072) | 1:1000 |

IF, immunofluorescence; CHGA, chromogranin A; TH, tyrosine hydroxylase; NES, nestin; VIM, vimentin.

**Supplementary Table 3**. Primer pairs for chromaffin cell markers, adrenomedullary stem/progenitor markers, neural markers, and reference genes.

| Target gene |  | Sequence (5’ 🡪 3’) |
| --- | --- | --- |
| *CHGA* | For | GCACATCAGCAGAAGAAACACA |
|  | Rev | TGGCTTCACCACTTTTCTCTG |
| *SYP* | For | CCGAGAGTGACCTCAGCATC |
|  | Rev | GAATTCGGCTGACGAGGAGT |
| *PNMT* | For | ACCTCCGCAACAACTACGC |
|  | Rev | GGCCTGAACCAATGTCGATG |
| *TH* | For | TCATGTCCCCGCGGTTCATT |
|  | Rev | TTCTCCTCAAAGGCCACAGC |
| *VIM* | For | ACGTCTTGACCTTGAACGCA |
|  | Rev | TCTTGGCAGCCACACTTTCA |
| *NES* | For | CTAGAGGAGGCAGGTGGTCT |
|  | Rev | CAAGGTGAAGGGGCATCACT |
| *SOX10* | For | TCCAGGCCCACTACAAGAGC |
|  | Rev | GTTGCCGAAGTCGATGTGAG |
| *GFAP* | For | CTCGCCGCTCCTACGTCT |
|  | Rev | GTAGCTGGCAAAGCGGTCA |
| *TUBB3* | For | CAACCAGATCGGGGCCAAGT |
|  | Rev | TGAAGAGATGTCCAAAGGCCC |
| *GAPDH* | For | TTCTTTTGCGTCGCCAGCC |
|  | Rev | CTTCCCGTTCTCAGCCTTGAC |
| *YWHAZ* | For | GGAGATAAAAAGAACATCCAGTCAT |
|  | Rev | TTCTCAGCACCTTCCGTCTT |
| *B2M* | For | AGTATGCCTGCCGTGTGAAC |
|  | Rev | ATGCGGCATCTTCAAACCTC |
| *GUSB* | For | TACGAACGGGAGGTGATCCT |
|  | Rev | TGGCGATAGTGATTCGGAGC |
| *HPRT1* | For | CCTGGCGTCGTGATTAGTGA |
|  | Rev | CGAGCAAGACGTTCAGTCCT |
| *EEF1A1* | For | GTTTGCCGCCAGAACACAGG |
|  | Rev | TGGACTTGCCCGAATCTACG |

For, forward primer; Rev, reverse primer; *CHGA*, chromogranin A; *SYP*, synaptophysin; *PNMT*, phenylethanolamine N-methyltransferase; *TH*, tyrosine hydroxylase; *VIM*, vimentin; *NES*, nestin; *SOX10*, SRY-box transcription factor 10; *GFAP*, glial fibrillary acidic protein; *TUBB3*, tubulin beta 3 class III; *GAPDH*, glyceraldehyde-3-phosphate dehydrogenase; *YWHAZ*, tyrosine 3-monooxygenase/tryptophan 5-monooxygenase activation protein zeta; *B2M*, beta-2-microglobulin; *GUSB*, glucuronidase beta; *HPRT1*, hypoxanthine-guanine phosphoribosyltransferase 1; *EEF1A1*, eukaryotic translation elongation factor 1 alpha 1

# Supplementary Figures

**Supplementary Figure 1.** Histological staining of a PCC dense cellular cluster showing signs of cellular degeneration.


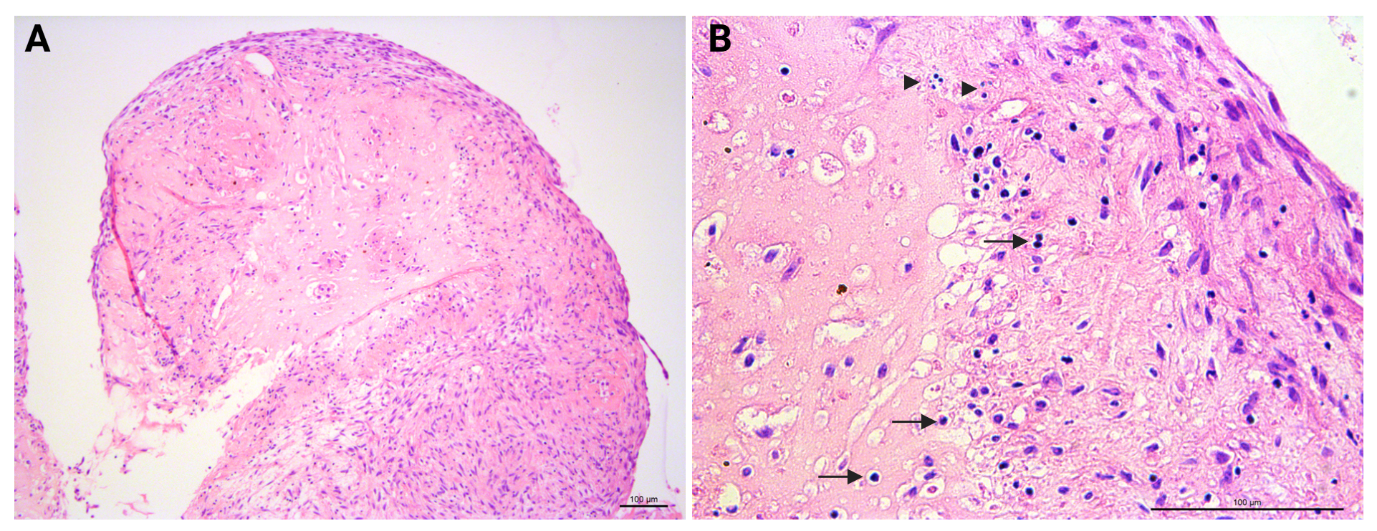


(A) Hematoxylin and eosin staining at 10x magnification of a dense cellular cluster; (B) 40x magnification showing pyknotic (arrows) and fragmented (arrowheads) nuclei interspersed within the extracellular matrix.
PCC, pheochromocytoma.
